# Editorial: Reactive Oxygen Species (ROS) Detection Methods in Biological System

**DOI:** 10.3389/fphys.2019.01316

**Published:** 2019-10-17

**Authors:** Ankush Prasad, Pavel Pospíšil, Mika Tada

**Affiliations:** ^1^Department of Biophysics, Centre of the Region Haná for Biotechnological and Agricultural Research, Faculty of Science, Palacký University, Olomouc, Czechia; ^2^Center for General Education, Tohoku Institute of Technology, Sendai, Japan; ^3^Biomedical Engineering Research Laboratory, Tohoku Institute of Technology, Sendai, Japan

**Keywords:** reactive oxygen species, oxidative stress, fluorescence imaging, proteomic signature, arsenic stress, drought

Metabolic processes such as respiration and oxidative stress are associated with the formation of reactive oxygen species (ROS). Any kind of fluctuation in environmental stress factors either biotic or abiotic is known to be linked with an increased level of ROS among which superoxide radical anion (O_2_^•−^); hydrogen peroxide (H_2_O_2_), hydroxyl radical (HO^•^), nitric oxide (NO), peroxynitrite (ONOO^−^), and singlet oxygen (^1^O_2_) have been widely studied (Kohno et al., 2008; Tada et al., 2009; Ichiishi et al., 2015; Zhang et al., 2018; Pospíšil et al., 2019). The ROS bear huge impact influencing the different cellular functions due to its involvement in the regulation of several cellular processes within a living system. However, because of its high reactivity, it is at the same time, harmful to the cell and is well-known to be associated with several biological dysfunction and diseases. Due to short life time and high reactivity, the detection of ROS in the biological system has always been very challenging. Different detection techniques and methodologies have been applied for quantification and localization of ROS and their reactive intermediates during the past decades both in *in vitro* and *in vivo* systems (Kohno et al., 2008; Tada et al., 2009; Ichiishi et al., 2015; Mattila et al., 2015). The detection is mainly based on:

- spectroscopic methods for instance, the use of specific spin traps and probes forming paramagnetic adducts.
- fluorescent-dependent methods where oxidant sensitive probes provide enhanced fluorescence under the condition of oxidative stress generation.
- chemiluminescent probes where monitoring of photon generation is monitored as a result of probes reaction with reactive species.
- spectrophotometric methods where reaction of reactive species with redox substances lead to change in absorbance.
- chromatographic method where separation and identification of reactive species and product are achieved.
- electrochemical sensors (such as chip-type biosensors) which is based on changes in oxidation/reduction current upon reactive species generation (Prasad et al., 2016).

The current Research Topic is aimed to bring contributions to the application of different methods for detection and localization of ROS and their reactive intermediates. We invited researchers who work on the cellular, systemic and molecular aspect of ROS to bring a group of experimental contributions on the application of different spectroscopic, microscopic and biochemical methods for the detection of reactive intermediates.

Three research articles show new findings and underscore the sophistication of the plant responses and its detection under environmental stimuli. In the study by Kumar et al., generation of ROS and subsequent protein radical formation in plants as a response to photooxidative stress has been demonstrated using electron paramagnetic resonance (EPR) spectroscopy, validated further using confocal laser scanning microscopy (CLSM), respectively. Furthermore, the characterization of protein radical was done using immuno-spin trapping. The use of immuno-spin trapping for protein radical detection is among the first of its type and can bring a new perspective toward understanding the role of protein radical for survival during photooxidative stress. Usage of fluorescent probes suffers limitations. Sedlárová and Luhová have attempted to provide a comprehensive protocol on working with CLSM and the precaution which should be taken into account while working with fluorescent probes. The authors have discussed the biological characteristics which should be taken into account as it can influence staining of samples thus providing false positive and negative results. Considering the fact that each method bears its drawback, the rational use of more than one method is recommended to conclude.

Niwano et al., discussed in the review the pro-oxidative properties of polyphenols and generation of highly reactive HO^•^ formation as a result of its photoirradiation. The authors provide a future perspective on the usage of grape pomace which is a by-product of wine-producing as a prooxidant (such as in the manufacturing of disinfectants and other sterilizing agents). Under metal stress, level of enzymatic antioxidant system/pigments was evaluated in plants by Alam et al. in the presence of stress relief substances. The antioxidant activity was further experimentally explored and is presented in the work of Iqbal et al., where the application of polyethylene glycol (PEG) was found to reduces the oxidative stress by upregulation of the enzymatic activities of the key enzymes such as peroxidase, superoxide dismutase, ascorbate peroxidase, glutathione reductase, and catalase. In the review by McDonagh, the author dealt with post-translational modifications (PTMs) induced by ROS. The mini-review summarizes the current proteomic approaches in the identification and quantification of ROS induced PTMs and its role in cellular signaling has been reviewed.

Altogether, the articles included in this Research Topic brings exciting results on *in vivo* and *in vitro* detection and localization of ROS; the shortcoming of the detection methods and the consideration that should be taken into account; its generation from endogenous pigments; the interplay between oxidative stress and antioxidants and PTM as proteomic signatures.

## Author Contributions

AP drafted the editorial. PP and MT read and modified the editorial. All authors approved it for publication.

### Conflict of Interest

The authors declare that the research was conducted in the absence of any commercial or financial relationships that could be construed as a potential conflict of interest.
